# 4-Cyano­anilinium bromide

**DOI:** 10.1107/S1600536812037014

**Published:** 2012-09-08

**Authors:** David J. Vumbaco, Michael N. Kammer, Lynn V. Koplitz, Joel T. Mague

**Affiliations:** a Department of Biological Sciences, Loyola University, New Orleans, LA 70118, USA; b Department of Physics, Loyola University, New Orleans, LA 70118, USA; c Department of Chemistry, Loyola University, New Orleans, LA 70118, USA; d Department of Chemistry, Tulane University, New Orleans, LA 70118, USA

## Abstract

In the crystal structure of the title compound, C_7_H_7_N_2_^+^·Br^−^, the cations are associated into inversion dimers through weak pairwise C—H⋯N hydrogen bonds. The dimers further form stepped sheets *via* weak pairwise C—H⋯N hydrogen bonds. In the sheets, the spacing between the mean planes of the laterally displaced aromatic rings in adjacent dimers is 1.124 (6) Å. Three N—H⋯Br inter­actions and two weak C—H⋯Br inter­actions per cation tie the sheets together.

## Related literature

For the structure of 4-cyano­anilinium choride, see: Colapietro *et al.* (1981 ▶). For the structure of 4-cyano­anilinium iodide, see: Mague *et al.* (2012 ▶). For the structure of anilinium bromide, see: Schweiss *et al.* (1983 ▶). For a discussion of C—H and N—H hydrogen bonding to halide ions, see: Steiner (1998 ▶).
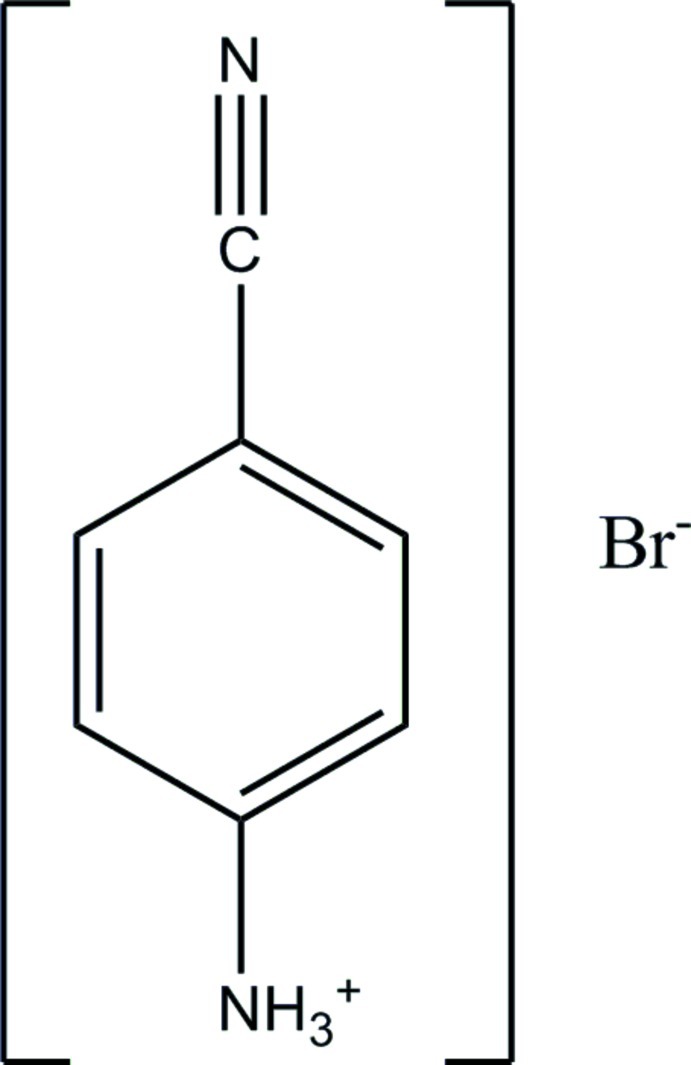


## Experimental

### 

#### Crystal data

C_7_H_7_N_2_^+^·Br^−^
*M* *_r_* = 199.06
Triclinic, 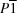

*a* = 4.3102 (10) Å
*b* = 6.1076 (13) Å
*c* = 14.510 (3) Å
α = 91.719 (3)°
β = 93.290 (3)°
γ = 101.428 (3)°
*V* = 373.46 (14) Å^3^
*Z* = 2
Mo *K*α radiation
μ = 5.42 mm^−1^
*T* = 100 K
0.20 × 0.19 × 0.16 mm

#### Data collection

Bruker SMART APEX CCD diffractometer
Absorption correction: numerical (*SADABS*; Sheldrick, 2009 ▶) *T*_min_ = 0.631, *T*_max_ = 0.837
6534 measured reflections
1874 independent reflections
1802 reflections with *I* > 2σ(*I*)
*R*_int_ = 0.032

#### Refinement

*R*[*F* ^2^ > 2σ(*F* ^2^)] = 0.020
*wR*(*F* ^2^) = 0.051
*S* = 1.06
1874 reflections
91 parameters
H-atom parameters constrained
Δρ_max_ = 0.86 e Å^−3^
Δρ_min_ = −0.41 e Å^−3^

### 

Data collection: *APEX2* (Bruker, 2010 ▶); cell refinement: *SAINT* (Bruker, 2009 ▶); data reduction: *SAINT*; program(s) used to solve structure: *SHELXM* (Sheldrick, 1998 ▶, 2004 ▶); program(s) used to refine structure: *SHELXL97* (Sheldrick, 2008 ▶); molecular graphics: *SHELXTL* (Sheldrick, 2008 ▶); software used to prepare material for publication: *SHELXTL*.

## Supplementary Material

Crystal structure: contains datablock(s) I, global. DOI: 10.1107/S1600536812037014/jj2147sup1.cif

Structure factors: contains datablock(s) I. DOI: 10.1107/S1600536812037014/jj2147Isup2.hkl

Supplementary material file. DOI: 10.1107/S1600536812037014/jj2147Isup3.cml

Additional supplementary materials:  crystallographic information; 3D view; checkCIF report

## Figures and Tables

**Table 1:** Hydrogen-bond geometry (Å, °)

*D*—H⋯*A*	*D*—H	H⋯*A*	*D*⋯*A*	*D*—H⋯*A*
N1—H1*A*⋯Br1	0.88	2.47	3.3209 (16)	162
C2—H2⋯Br1^i^	0.95	2.87	3.7316 (18)	151
C3—H3⋯N2^ii^	0.95	2.62	3.466 (2)	149
C5—H5⋯N2^iii^	0.95	2.69	3.517 (2)	146
C6—H6⋯Br1^iv^	0.95	3.00	3.8063 (18)	144
N1—H1*B*⋯Br1^iv^	0.88	2.54	3.4174 (16)	175
N1—H1*C*⋯Br1^v^	0.88	2.49	3.3400 (16)	162

Symmetry codes: (i) 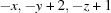; (ii) 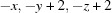; (iii) 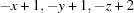; (iv) 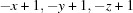; (v) 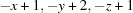.

**Fig. 1.**
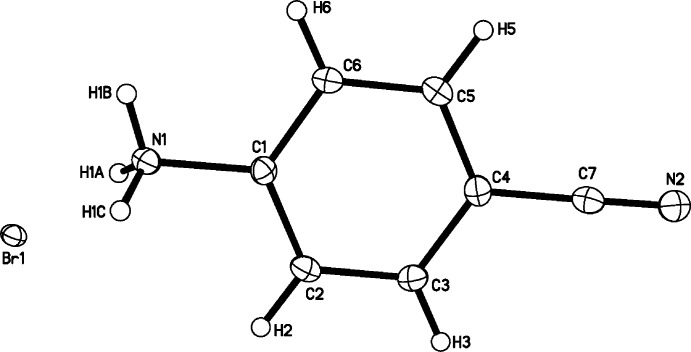
Perspective view of the asymmetric unit with displacement ellipsoids drawn at the 50% probability level

**Fig. 2.**
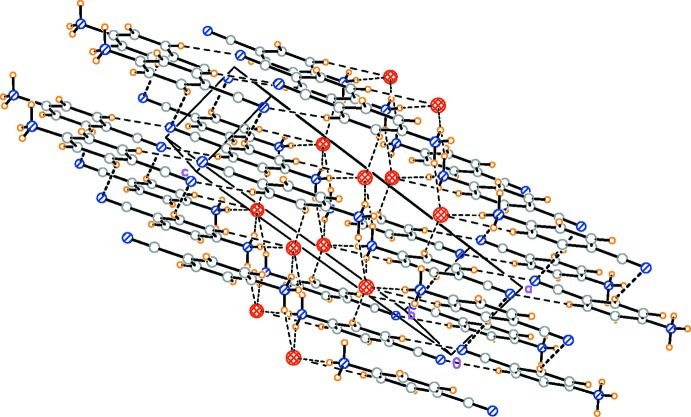
Packing showing the stepped layer structure. N—H···Br, C—H···N and C—H···Br interactions are shown as dashed lines. Color key: C = gray, H = orange, Br = red, N = blue.

**Fig. 3.**
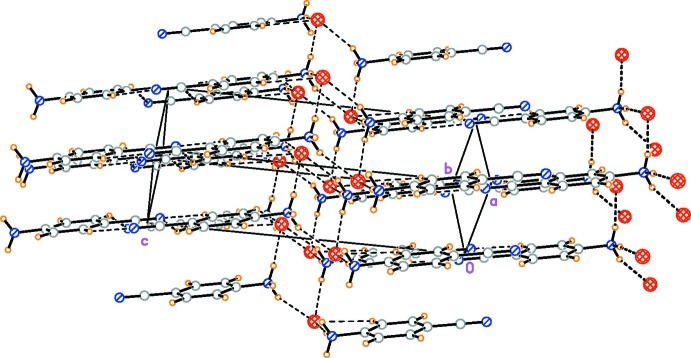
Packing showing the rectangular channels. N—H···Br, C—H···N and C—H···Br interactions are shown as dashed lines. Color key: C = gray, H = orange, Br = red, N = blue.

## supplementary crystallographic information

### Comment

In the title compound, [C_7_H_7_N_2_]^+^ Br^-^, the cations are associated into dimers through weak, pairwise C3—H3···N2 intermolecular interactions (Fig. 1). The dimers further form stepped sheets *via* weak, pairwise C5—H5···N2 intermolecular interactions. In these sheets the spacing between the mean planes of the aromatic rings in adjacent dimers is 1.124 (6) Å (Table 1). The three hydrogen atoms of the anilinium group make contacts with the surrounding anions of 2.47 - 2.54 Å. These distances compare well with the mean value of 2.49 (2) Å for an N^+^—H···Br^-^ hydrogen bond (Steiner, 1998) and serve, together with weak C2—H2···Br1 and C6—H6···Br1 interactions, to tie the stepped sheets into a layer structure (Fig. 2) with the layers 3.493 (7) Å apart and forming rectangular channels of width *ca* 12.8 Å (Fig. 3).

### Experimental

0.55 g of 4-cyanoaniline and 2.5 ml of aquous hydrobromic acid (2 *M*) were combined in 10 ml of ethanol. This solution was slowly evaporated to dryness under ambient conditions to form crystals of the title compound.

### Refinement

H-atoms attached to C were placed in calculated positions (C—H = 0.95 - 0.98 Å) while those attached to N were placed in sites determined from a difference map and their coordinates adjusted to give N—H = 0.88 Å. All H-atoms were included as riding contributions with isotropic displacement parameters 1.2 times those of the attached atoms.

### Crystal data

**Table:**

C_7_H_7_N_2_^+^·Br^−^	*Z* = 2
*M**_r_* = 199.06	*F*(000) = 196
Triclinic, *P*1	*D*_x_ = 1.770 Mg m^−^^3^
Hall symbol: -P 1	Mo *K*α radiation, λ = 0.71073 Å
*a* = 4.3102 (10) Å	Cell parameters from 5589 reflections
*b* = 6.1076 (13) Å	θ = 2.8–29.1°
*c* = 14.510 (3) Å	µ = 5.42 mm^−^^1^
α = 91.719 (3)°	*T* = 100 K
β = 93.290 (3)°	Block, colourless
γ = 101.428 (3)°	0.20 × 0.19 × 0.16 mm
*V* = 373.46 (14) Å^3^	

### Data collection

**Table:**

Bruker SMART APEX CCD diffractometer	1874 independent reflections
Radiation source: fine-focus sealed tube	1802 reflections with *I* > 2σ(*I*)
Graphite monochromator	*R*_int_ = 0.032
φ and ω scans	θ_max_ = 29.2°, θ_min_ = 2.8°
Absorption correction: numerical (*SADABS*; Sheldrick, 2009)	*h* = −5→5
*T*_min_ = 0.631, *T*_max_ = 0.837	*k* = −8→8
6534 measured reflections	*l* = −19→19

### Refinement

**Table:**

Refinement on *F*^2^	Primary atom site location: structure-invariant direct methods
Least-squares matrix: full	Secondary atom site location: difference Fourier map
*R*[*F*^2^ > 2σ(*F*^2^)] = 0.020	Hydrogen site location: inferred from neighbouring sites
*wR*(*F*^2^) = 0.051	H-atom parameters constrained
*S* = 1.06	*w* = 1/[σ^2^(*F*_o_^2^) + (0.0248*P*)^2^ + 0.1891*P*] where *P* = (*F*_o_^2^ + 2*F*_c_^2^)/3
1874 reflections	(Δ/σ)_max_ = 0.002
91 parameters	Δρ_max_ = 0.86 e Å^−^^3^
0 restraints	Δρ_min_ = −0.41 e Å^−^^3^

### Special details

**Table:**

Experimental. The diffraction data were obtained from 3 sets of 400 frames, each of width 0.5 °. in omega, collected at phi = 0.00, 90.00 and 180.00 °. and 2 sets of 800 frames, each of width 0.45 ° in phi, collected at omega = -30.00 and 210.00 °. The scan time was 10 sec/frame.
Geometry. All e.s.d.'s (except the e.s.d. in the dihedral angle between two l.s. planes) are estimated using the full covariance matrix. The cell e.s.d.'s are taken into account individually in the estimation of e.s.d.'s in distances, angles and torsion angles; correlations between e.s.d.'s in cell parameters are only used when they are defined by crystal symmetry. An approximate (isotropic) treatment of cell e.s.d.'s is used for estimating e.s.d.'s involving l.s. planes.
Refinement. Refinement of *F*^2^ against ALL reflections. The weighted *R*-factor *wR* and goodness of fit *S* are based on *F*^2^, conventional *R*-factors *R* are based on *F*, with *F* set to zero for negative *F*^2^. The threshold expression of *F*^2^ > σ(*F*^2^) is used only for calculating *R*-factors(gt) *etc*. and is not relevant to the choice of reflections for refinement. *R*-factors based on *F*^2^ are statistically about twice as large as those based on *F*, and *R*- factors based on ALL data will be even larger. H-atoms attached to carbon were placed in calculated positions (C—H = 0.95 Å) while those attached to nitrogen were placed in locations derived from a difference map and then their coordinates adjusted to give an N—H distance of 0.88 Å. All were included as riding contributions with isotropic displacement parameters 1.2 times those of the attached atoms.

### Fractional atomic coordinates and isotropic or equivalent isotropic displacement parameters (Å^2^)

**Table:**

	*x*	*y*	*z*	*U*_iso_*/*U*_eq_	
Br1	0.08172 (3)	0.73901 (2)	0.423553 (10)	0.01208 (7)	
N1	0.5934 (3)	0.7244 (2)	0.60221 (10)	0.0128 (3)	
H1A	0.4298	0.7029	0.5615	0.015*	
H1B	0.6868	0.6102	0.5940	0.015*	
H1C	0.7187	0.8527	0.5922	0.015*	
N2	0.1311 (4)	0.7765 (3)	1.04130 (11)	0.0215 (3)	
C1	0.4868 (4)	0.7327 (3)	0.69615 (11)	0.0117 (3)	
C2	0.3482 (4)	0.9098 (3)	0.72253 (12)	0.0142 (3)	
H2	0.3199	1.0208	0.6801	0.017*	
C3	0.2514 (4)	0.9219 (3)	0.81193 (12)	0.0148 (3)	
H3	0.1545	1.0409	0.8312	0.018*	
C4	0.2980 (4)	0.7575 (3)	0.87320 (12)	0.0140 (3)	
C5	0.4367 (4)	0.5796 (3)	0.84545 (12)	0.0158 (3)	
H5	0.4650	0.4679	0.8875	0.019*	
C6	0.5326 (4)	0.5674 (3)	0.75594 (12)	0.0142 (3)	
H6	0.6278	0.4481	0.7361	0.017*	
C7	0.2031 (4)	0.7694 (3)	0.96694 (13)	0.0168 (3)	

### Atomic displacement parameters (Å^2^)

**Table:**

	*U*^11^	*U*^22^	*U*^33^	*U*^12^	*U*^13^	*U*^23^
Br1	0.01341 (9)	0.01043 (10)	0.01308 (10)	0.00377 (6)	0.00081 (6)	0.00256 (6)
N1	0.0141 (6)	0.0118 (7)	0.0130 (7)	0.0032 (5)	0.0006 (5)	0.0018 (5)
N2	0.0304 (9)	0.0179 (8)	0.0179 (8)	0.0073 (7)	0.0055 (7)	0.0020 (6)
C1	0.0118 (7)	0.0123 (8)	0.0102 (7)	0.0013 (6)	−0.0010 (6)	−0.0002 (6)
C2	0.0159 (8)	0.0124 (8)	0.0149 (8)	0.0040 (6)	0.0000 (6)	0.0033 (6)
C3	0.0167 (8)	0.0127 (8)	0.0157 (8)	0.0050 (7)	0.0009 (6)	0.0001 (6)
C4	0.0141 (8)	0.0150 (8)	0.0122 (8)	0.0017 (6)	0.0007 (6)	0.0008 (6)
C5	0.0185 (8)	0.0143 (8)	0.0152 (8)	0.0047 (7)	0.0007 (6)	0.0038 (6)
C6	0.0165 (8)	0.0118 (8)	0.0152 (8)	0.0045 (6)	0.0008 (6)	0.0018 (6)
C7	0.0201 (8)	0.0125 (8)	0.0181 (9)	0.0038 (7)	0.0011 (7)	0.0026 (6)

### Geometric parameters (Å, º)

**Table:**

N1—C1	1.466 (2)	C2—H2	0.9500
N1—H1A	0.8800	C3—C4	1.397 (2)
N1—H1B	0.8801	C3—H3	0.9500
N1—H1C	0.8800	C4—C5	1.399 (2)
N2—C7	1.142 (3)	C4—C7	1.447 (2)
C1—C6	1.387 (2)	C5—C6	1.390 (2)
C1—C2	1.389 (2)	C5—H5	0.9500
C2—C3	1.390 (2)	C6—H6	0.9500
			
C1—N1—H1A	110.3	C2—C3—H3	120.3
C1—N1—H1B	110.7	C4—C3—H3	120.3
H1A—N1—H1B	106.0	C3—C4—C5	120.97 (16)
C1—N1—H1C	108.9	C3—C4—C7	120.11 (16)
H1A—N1—H1C	108.7	C5—C4—C7	118.91 (16)
H1B—N1—H1C	112.2	C6—C5—C4	119.56 (16)
C6—C1—C2	122.32 (16)	C6—C5—H5	120.2
C6—C1—N1	119.28 (15)	C4—C5—H5	120.2
C2—C1—N1	118.38 (15)	C1—C6—C5	118.79 (16)
C1—C2—C3	118.97 (16)	C1—C6—H6	120.6
C1—C2—H2	120.5	C5—C6—H6	120.6
C3—C2—H2	120.5	N2—C7—C4	178.9 (2)
C2—C3—C4	119.38 (16)		
			
C6—C1—C2—C3	−0.1 (3)	C7—C4—C5—C6	−179.23 (17)
N1—C1—C2—C3	−178.82 (15)	C2—C1—C6—C5	−0.1 (3)
C1—C2—C3—C4	0.5 (3)	N1—C1—C6—C5	178.66 (15)
C2—C3—C4—C5	−0.8 (3)	C4—C5—C6—C1	−0.2 (3)
C2—C3—C4—C7	179.08 (16)	C3—C4—C7—N2	−162 (11)
C3—C4—C5—C6	0.7 (3)	C5—C4—C7—N2	18 (12)

### Hydrogen-bond geometry (Å, º)

**Table:**

*D*—H···*A*	*D*—H	H···*A*	*D*···*A*	*D*—H···*A*
N1—H1*A*···Br1	0.88	2.47	3.3209 (16)	162
C2—H2···Br1^i^	0.95	2.87	3.7316 (18)	151
C3—H3···N2^ii^	0.95	2.62	3.466 (2)	149
C5—H5···N2^iii^	0.95	2.69	3.517 (2)	146
C6—H6···Br1^iv^	0.95	3.00	3.8063 (18)	144
N1—H1*B*···Br1^iv^	0.88	2.54	3.4174 (16)	175
N1—H1*C*···Br1^v^	0.88	2.49	3.3400 (16)	162

Symmetry codes: (i) −*x*, −*y*+2, −*z*+1; (ii) −*x*, −*y*+2, −*z*+2; (iii) −*x*+1, −*y*+1, −*z*+2; (iv) −*x*+1, −*y*+1, −*z*+1; (v) −*x*+1, −*y*+2, −*z*+1.

## References

[bb1] Bruker (2009). *SAINT* Bruker AXS Inc., Madison, Wisconsin, USA.

[bb2] Bruker (2010). *APEX2* Bruker AXS Inc., Madison, Wisconsin, USA.

[bb3] Colapietro, M., Domenicano, A., Marciante, C. & Portalone, G. (1981). *Acta Cryst.* B**37**, 387–394.

[bb4] Mague, J. T., Vumbaco, D. J., Kammer, M. N. & Koplitz, L. V. (2012). *Acta Cryst.* E**68**, o2623. 10.1107/S1600536812033466 PMC3435651 22969524

[bb5] Schweiss, B. P., Fuess, H., Fecher, G. & Weiss, A. (1983). *Z. Naturforsch. Teil A*, **38**, 350–358.

[bb10] Sheldrick, G. M. (1998). *SHELX: applications to macromolecules.* In *Direct Methods for Solving Macromolecular Structures* edited by S. Fortier, pp. 401–411. Dordrecht: Kluwer Academic Publishers.

[bb6] Sheldrick, G. M. (2004). *SHELXM* University of Göttingen, Germany.

[bb7] Sheldrick, G. M. (2008). *Acta Cryst.* A**64**, 112–122. 10.1107/S0108767307043930 18156677

[bb8] Sheldrick, G. M. (2009). *SADABS* University of Göttingen, Germany.

[bb9] Steiner, T. (1998). *Acta Cryst.* B**54**, 456–463.

